# Gene co-expression network reveals shared modules predictive of stage and grade in serous ovarian cancers

**DOI:** 10.18632/oncotarget.17785

**Published:** 2017-05-11

**Authors:** Qian Sun, Haiyue Zhao, Cong Zhang, Ting Hu, Jianli Wu, Xingguang Lin, Danfeng Luo, Changyu Wang, Li Meng, Ling Xi, Kezhen Li, Junbo Hu, Ding Ma, Tao Zhu

**Affiliations:** ^1^ Cancer Biology Research Center, Key Laboratory of the Ministry of Education, Tongji Hospital, Tongji Medical College, Huazhong University of Science and Technology, Wuhan, People's Republic of China

**Keywords:** ovarian cancer, WGCNA, gene co-expression network, grade, stage

## Abstract

Serous ovarian cancer (SOC) is the most lethal gynecological cancer. Clinical studies have revealed an association between tumor stage and grade and clinical prognosis. Identification of meaningful clusters of co-expressed genes or representative biomarkers related to stage or grade may help to reveal mechanisms of tumorigenesis and cancer development, and aid in predicting SOC patient prognosis. We therefore performed a weighted gene co-expression network analysis (WGCNA) and calculated module-trait correlations based on three public microarray datasets (GSE26193, GSE9891, and TCGA), which included 788 samples and 10402 genes. We detected four modules related to one or more clinical features significantly shared across all modeling datasets, and identified one stage-associated module and one grade-associated module. Our analysis showed that MMP2, COL3A1, COL1A2, FBN1, COL5A1, COL5A2, and AEBP1 are top hub genes related to stage, while CDK1, BUB1, BUB1B, BIRC5, AURKB, CENPA, and CDC20 are top hub genes related to grade. Gene and pathway enrichment analyses of the regulatory networks involving hub genes suggest that extracellular matrix interactions and mitotic signaling pathways are crucial determinants of tumor stage and grade. The relationships between gene expression modules and tumor stage or grade were validated in five independent datasets. These results could potentially be developed into a more objective scoring system to improve prediction of SOC outcomes.

## INTRODUCTION

Epithelial ovarian cancer (EOC) is the most lethal gynecological cancer and the fifth most common cause of cancer-related death among women in the United States [1]. Serous ovarian cancer (SOC), peritoneal carcinoma, carcinosarcoma, and mixed carcinoma with serous component account for 78% of all cases and 87% of advanced stage cases of EOC [2]. SOC is the most common histological subtype of EOC. Due to latent symptoms and lack of reliable early screening methods, most SOCs are diagnosed at an advanced stage (stage III-IV; International Federation of Gynecology and Obstetrics, FIGO) [3]. As advanced-stage or high-grade SOCs are more likely to have a poor prognosis [4], discovering gene expression signatures associated with SOC stage and grade outcomes is crucial.

Previous integrated genomic analyses of ovarian carcinoma subdivided SOCs into multiple molecular subtypes and attempted to explain their association with prognosis [5–7]. However, some of the SOC subtypes proposed by Tothill et al. were mixed with endometrioid ovarian cancers [6], while those defined by Verhaak et al. contained only high-grade SOCs [7]. In the above studies, researchers established tumor subtypes based on inherent gene expression profiles and then explored their relationship to clinical features, but few direct correlations were detected. From a clinical point of view, an applicable subtype system based on gene-related prognosis that can guide clinical therapeutic strategies is desirable. Recent advances in gene interaction network methodologies encouraged researchers to investigate possible intrinsic links between functional gene clusters (i.e. functional modules) and prognostic factors. Identification of meaningful modules related to grade and stage could be beneficial for inferring tumor mechanism, predicting patient survival, and establishing novel diagnostic or therapeutic targets. A weighted gene co-expression network analysis (WGCNA) was proposed to reconstruct robust gene co-expression networks (modules). These modules were constructed in terms of large-scale gene expression profiles and the distinction of centrally located genes (hub genes) that drive key cellular signaling pathways [8, 9]. The WGCNA approach has provided functional interpretation tools in systems biology and led to new insights into the pathophysiology of breast cancer and endometrial cancer [9–14]. Although WGCNA has been applied to detect TP53 missense or null mutations in ovarian cancer [15], there are no reports applying WGCNA to systematically identify gene co-expression networks associated with clinical-pathological factors in SOCs.

To fulfill this gap, we conducted a WGCNA and calculated module-trait correlations based on three public microarray datasets (GSE26193, GSE9891, and TCGA), which included 788 samples and 10402 genes. This approach identified meaningful co-expression modules significantly related to tumor grade and stage, and revealed hub genes contributing to extracellular matrix interactions and mitosis in SOC. Our study provides a novel and broad application platform for the identification of SOC gene signatures, and may be useful to characterize new molecular targets and develop effective therapeutic strategies.

## RESULTS

### Construction of gene co-expression network

**Figure 1:**
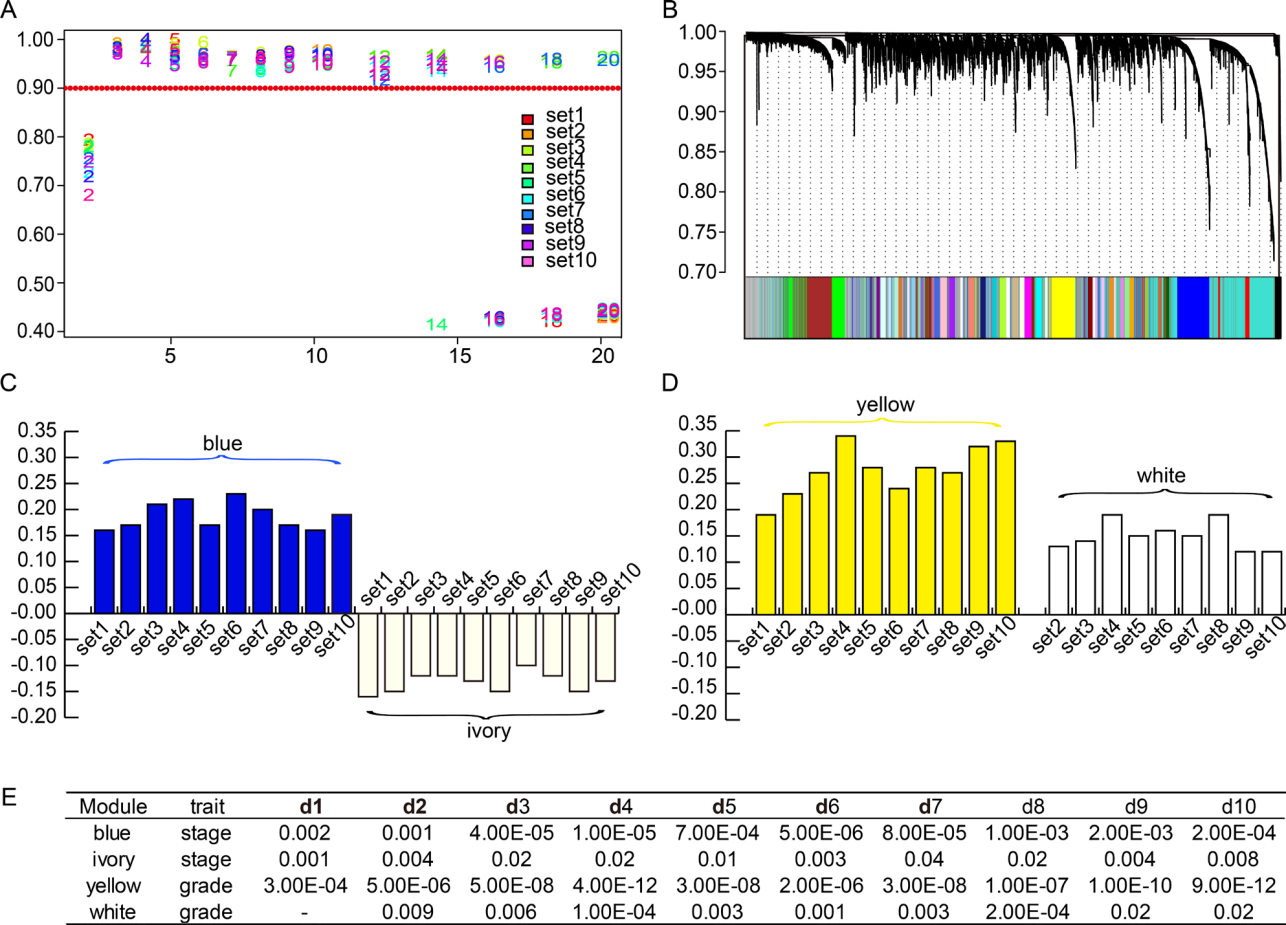
Weighted gene co-expression network of SOC (**A**) Network topology analysis was employed to choose a soft-thresholding power to achieve scale-free topology in all modeling sets. (**B**) Fifty-four significant co-expression gene modules shared in ten random sampling sets were detected with WGCNA. Consensus gene dendrogram and module colors denote correspondence. (**C**) Correlation values of blue and ivory module-trait relationships across ten random sampling datasets. (**D**) Correlation values of yellow and white module-trait relationships across ten random sampling datasets. (**E**) *P* values of module-trait relationships of two stage-associated and two grade-associated modules across ten random sampling datasets (*p* < 0.05).

WGCNA was performed to identify gene co-expression networks associated with SOC clinical-pathological factors. Three SOC datasets, namely, GSE26193, GSE9891, and TCGA, were adopted from the curatedOvarianData Bioconductor package (Table 1) [16, 17]. In total, 788 samples and 10402 genes were included, and ten arbitrary datasets, each containing 50% of all samples, were built through random sampling. Gene co-expression networks were then built among these ten datasets (d1 to d10). As 3 is the lowest value that allows achieving more than 90% similarities in topology models of ten datasets (Figure 1A), a soft threshold of 3 was implemented, resulting in the detection of 54 significant modules (Figure 1B).

**Table 1:** General information of involved three modeling and five validation datasets

datasets	platform	Involved samples	Grade (I/II/III)	Stage (I/II/III/IV)	Recurrence Status (N/Y)	Vital status (N/Y)
**Modeling datasets**						
GSE26193	hgu133plus2	79	4/19/56	11/6/48/14	16/63	19/60
GSE9891	hgu133plus2	237	7/86/144	11/9/195/21	62/175	126/111
TCGA	hthgu133a	472	6/58/408	13/24/365/68	225/247	218/254
**Validation datasets**						
GSE17260	hgug4112a	110	26/41/43	0/0/93/17	34/76	64/46
TCGA.RNASeqV2	RNASeq	261	1/28/226	0/18/209/33	123/138	114/143
GSE20565	hgu133plus2	140	6/27/63	18/9/52/15	-	-
PMID15897565	hgu133a	63	2/35/25	7/4/48/4	-	-
GSE49997	ABI Human Genome Survey Microarray V2	204	0/50/143	0/9/154/31	70/124	137/57

### Calculation of module-trait correlations in SOCs

For each module, we calculated correlations between gene expression and clinical features such as tumor stage, grade, recurrence time, vital time, recurrence status, and vital status. The last four features were regarded as prognostic traits. Consensus module-trait relationships across the ten sets were also presented as mutually significant correlations (*p* < 0.05). We noticed that there were multiple modules associated with one or more traits. In particular, there were consistent correlations among the ten sets in four modules, each named after their representative color: blue, ivory, yellow, and white. For instance, the blue and the ivory modules were related to tumor stage; the yellow module was related to grade; and the white module was related to grade in nine out of ten sets. Besides, correlations between gene expression patterns and prognostic traits were found in a minority of the ten sets. In short, two stage-associated and two grade-associated gene modules were identified in SOCs using WGCNA. The correlation indexes are shown in Supplementary Figure 1, and the significance of module-trait relationships is shown in Figure 1C–1E).

### Module preservation analysis

A summarized Z score was calculated to determine universal module preservation using WGCNA R software. Modules with a Z score > 10 were regarded as highly preserved. As recommended by the WGCNA author, all uncharacterized genes were assigned to the gray module, which should have a Z score lower than that of most other modules [18]. We could assert that 36/54 modules were highly conserved (Supplementary Table 1). The Z scores of the gray module and the four stage-associated or grade-associated modules were 78.82 (blue), 61.51 (yellow), 30.3 (white), 30.0 (gray) and 27.41 (ivory). The blue module was regarded as a representative stage-associated module and the yellow module as a grade-associated module, because they both contained higher conservation and consistent association with stage or grade. Supplementary Table 2 contains gene symbols inside these four modules.

### Identification of universal hub genes in the blue and yellow modules

The blue and yellow modules comprised 884 and 561 genes, respectively. Genes with the top 200 strongest connections within the blue and yellow modules from each set were extracted to show their connections and identify hub genes (Supplementary Figures 2 and 3). Within each network, node sizes, font sizes, and color depth are proportional to their connectivity (sum of in-module degrees). Shared hub genes were readily discernible in all ten sets.

**Figure 2:**
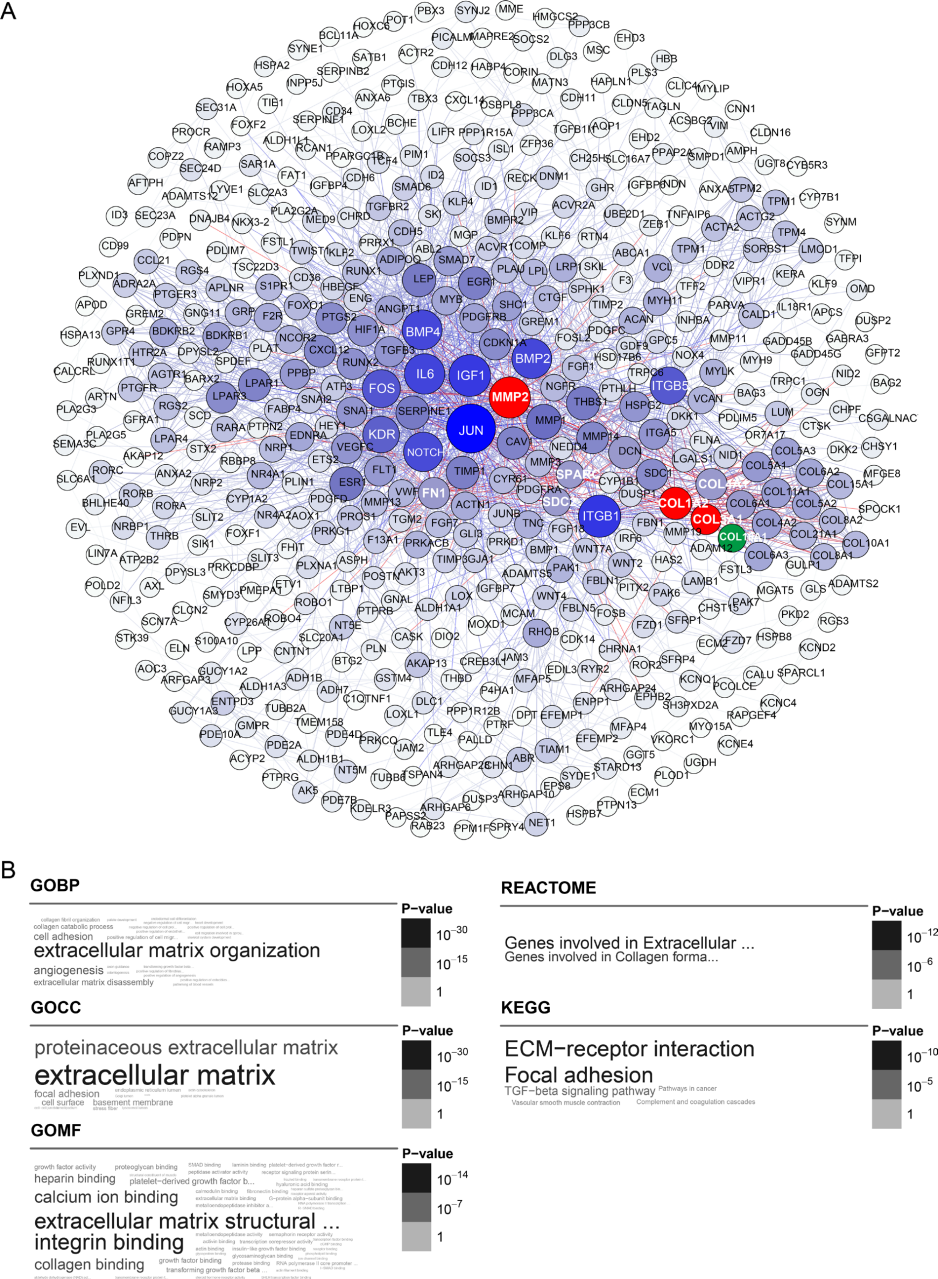
Blue module gene network and enrichment analysis (**A**) Top hub genes of the blue module are shown in blue; gene importance was assigned according to circle diameter and color depth, in descending order. Intersection of the top 25 hub genes with the high-quality STRING network is shown in red. COL16A1 is shown in green as a node connected to MMP2 and COL. (**B**) Gene ontology and pathway enrichment analysis of blue module genes.

**Figure 3:**
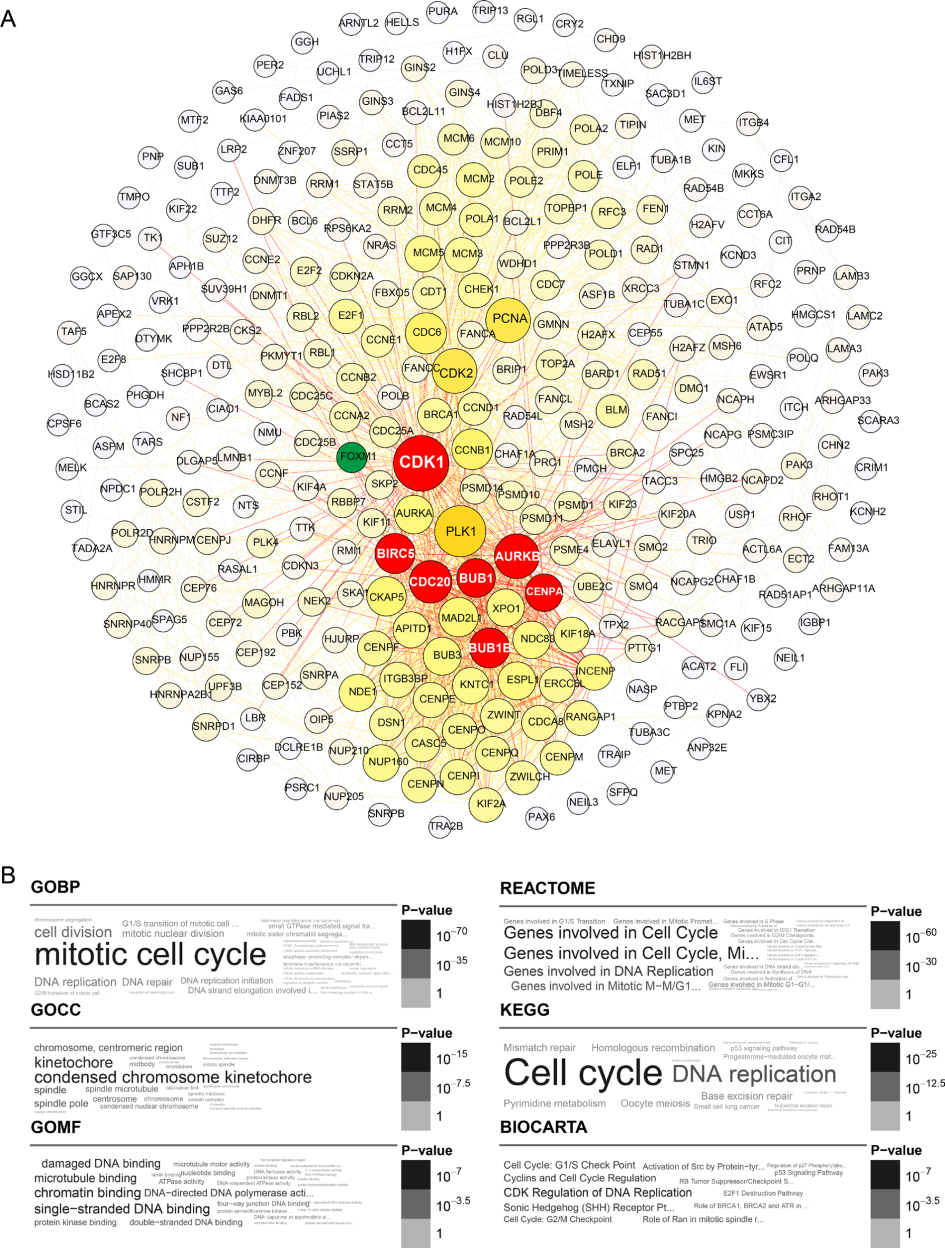
Yellow module gene network and enrichment analysis (**A**) Top hub genes of the yellow module are shown in yellow; gene importance was assigned according to circle diameter and color depth, in descending order. Intersection of the top 25 hub genes with the high-quality STRING network is shown in red. FOXM1 is shown in green as a node connected to CDK1 and CENPA. (**B**) Gene ontology and pathway enrichment analysis of yellow module genes.

To compare and integrate our gene co-expression networks with protein interaction data, we extracted a high-quality protein interaction network from the Search Tool for the Retrieval of Interacting Genes (STRING), which only contains interactions with a combined score above 600. The retrieved STRING network contained 16771 nodes and 392611 edges. Nodes were defined as individual genes in the network, and edges were defined as the interactions between genes. Subsequently, we found mutual genes in each module and in the STRING network gene set and extracted them from the respective subnetworks. As shown in Figures 2A and 3A, the blue module subnetwork contained 505 nodes and 2093 edges, while the yellow module subnetwork contained 313 nodes and 2215 edges. Since the subnetworks were extracted from a high-quality STRING protein interaction database section, derived from traceable interaction experiments, the data suggest that a tight regulatory relationship exists for these module genes in nature.

A comparison of the top 25 hub genes throughout the co-expression network among the ten datasets, and mutual subnetwork genes, is summarized in Table 2 (blue module) and Table 3 (yellow module). In the blue module co-expression network, MMP2, COL1A2, and COL3A1 were hub genes with tight relationships. MMP2 interacted with COL1A2 or COL3A1 through COL16A1 (Figure 2A). In the yellow module co-expression network, CDK1 interacted with CENPA through FOXM1, while CENPA, CDC20, AURKB, BUB1, BUB1B, and BIRC5 interacted with each other directly (Figure 3A). The regulatory networks among these hub genes, although complex, were organized in a similar topology.

**Table 2:** The top 25 hub genes of the blue module through ten datasets and the high-quality STRING subnetwork

Number	d1	d2	d3	d4	d5	d6	d7	d8	d9	d10	STRING_600
1	FBN1	FBN1	FBN1	FBN1	FBN1	FBN1	FBN1	FBN1	FBN1	FBN1	JUN
2	COL5A1	COL5A1	COL5A1	COL5A2	COL5A1	COL5A1	COL5A1	COL5A2	COL5A2	COL5A2	BMP4
3	COL5A2	AEBP1	AEBP1	COL5A1	COL5A2	COL5A2	COL5A2	COL5A1	COL5A1	COL5A1	BMP2
4	AEBP1	COL5A2	COL5A2	AEBP1	AEBP1	AEBP1	AEBP1	AEBP1	AEBP1	AEBP1	**MMP2**
5	FAP	SPARC	INHBA	SPARC	FAP	FAP	SNAI2	SPARC	FAP	SPARC	ITGB1
6	SPARC	SNAI2	FAP	FAP	SPARC	**MMP2**	FAP	FAP	SNAI2	FAP	FOS
7	**MMP2**	**MMP2**	SPARC	**MMP2**	INHBA	SNAI2	SPARC	INHBA	SPARC	**MMP2**	NOTCH1
8	SNAI2	FAP	COL3A1	INHBA	**MMP2**	CTSK	INHBA	**MMP2**	INHBA	INHBA	IGF1
9	INHBA	INHBA	SNAI2	SNAI2	CTSK	INHBA	**MMP2**	SNAI2	**MMP2**	SNAI2	KDR
10	VCAN	ADAM12	**MMP2**	VCAN	SNAI2	SPARC	CTSK	VCAN	VCAN	CTSK	IL6
11	CRISPLD2	CTSK	CTSK	CTSK	COL3A1	VCAN	LOX	CTSK	CTSK	COL3A1	FN1
12	COL3A1	VCAN	VCAN	COL3A1	VCAN	COL3A1	COL3A1	COL3A1	COL3A1	VCAN	CAV1
13	ADAM12	COL3A1	CRISPLD2	ADAM12	ADAM12	CRISPLD2	VCAN	ADAM12	ACTA2	COL11A1	THBS1
14	CTSK	COPZ2	ADAM12	**COL1A2**	CRISPLD2	ADAM12	ADAM12	CRISPLD2	CRISPLD2	ZEB1	**COL1A2**
15	LOX	COL6A3	COL6A3	COL6A3	COL11A1	COL6A3	PDLIM3	**COL1A2**	ADAM12	LOX	EGR1
16	**COL1A2**	CRISPLD2	LOX	CRISPLD2	LOX	ZEB1	CRISPLD2	COL6A3	PDLIM3	ADAM12	ESR1
17	COL6A3	**COL1A2**	COPZ2	CDH11	SERPINF1	LOX	SERPINF1	BGN	COPZ2	ACTA2	ITGB5
18	COL11A1	SERPINF1	COL11A1	ACTA2	ECM1	**COL1A2**	ACTA2	LOX	**COL1A2**	CRISPLD2	MMP1
19	CDH11	BGN	ACTA2	SERPINF1	LRRC15	COL11A1	COL6A3	SERPINF1	ZEB1	COL6A3	SERPINE1
20	PDLIM3	CDH11	PDLIM3	COL11A1	ANGPTL2	SERPINF1	ECM1	OLFML2B	COL6A3	PDLIM3	COL3A1
21	ZEB1	ZEB1	CDH11	PDLIM3	COL6A3	GLT8D2	GLT8D2	CDH11	COL11A1	CDH11	MMP14
22	COL6A2	LOX	LRRC15	ECM1	PDLIM3	ECM1	COL11A1	FN1	SERPINF1	COL10A1	DCN
23	SERPINF1	COL11A1	FN1	OLFML2B	**COL1A2**	COPZ2	COPZ2	COL11A1	LOX	SERPINF1	COL4A1
24	LHFP	PDLIM3	PDGFRB	LRRC15	COL10A1	CDH11	**COL1A2**	ACTA2	ECM1	**COL1A2**	SHC1
25	ANGPTL2	EDNRA	ECM1	GLT8D2	ACTA2	PDLIM3	LHFP	PDLIM3	CDH11	ECM1	HIF1A

d1–d10: ten datasets sampled from the modeling datasets.

**Table 3:** The top 25 hub genes of the yellow module through ten datasets and the high-quality STRING subnetwork

number	multi1	multi2	multi3	multi4	multi5	multi6	multi7	multi8	multi9	multi10	STRING_600
1	TPX2	TPX2	TPX2	KIF4A	TPX2	TPX2	TPX2	KIF4A	KIF4A	KIF4A	CDK1
2	CENPA	KIF4A	CENPA	CENPA	**BUB1**	KIF4A	CENPA	CENPA	CENPA	TPX2	PLK1
3	DLGAP5	CENPA	KIF4A	TPX2	KIF4A	NUSAP1	DLGAP5	TPX2	TPX2	NUSAP1	PCNA
4	KIF4A	**BUB1**	MELK	**BUB1**	RACGAP1	KIF15	KIF4A	**BUB1**	**BUB1**	CENPA	CDK2
5	**BUB1**	MELK	**BUB1**	UBE2C	DLGAP5	HJURP	**BUB1**	NUSAP1	DLGAP5	MELK	CCNB1
6	NUSAP1	DLGAP5	NUSAP1	MELK	MELK	**BUB1**	UBE2C	KIF15	MELK	**BUB1**	CDC20
7	KIF15	NUSAP1	HJURP	RACGAP1	UBE2C	CENPA	NUSAP1	DLGAP5	RACGAP1	KIF15	AURKB
8	NCAPH	UBE2C	RACGAP1	NCAPH	CENPA	NCAPH	KIF15	UBE2C	KIF15	DLGAP5	AURKA
9	UBE2C	ASPM	ASPM	KIF15	NUSAP1	MELK	NCAPH	MELK	HJURP	CCNB2	MAD2L1
10	RACGAP1	HJURP	NCAPH	ASPM	KIF15	DLGAP5	MELK	RACGAP1	UBE2C	HJURP	MCM5
11	ASPM	KIF15	DLGAP5	CCNB2	KIF23	RACGAP1	CCNB2	HJURP	CCNB2	BIRC5	CDC6
12	HJURP	CCNB2	KIF15	HJURP	BIRC5	UBE2C	HJURP	ASPM	ASPM	RACGAP1	BUB1
13	KIF23	RACGAP1	**BUB1B**	DLGAP5	**BUB1B**	ASPM	RACGAP1	**BUB1B**	NUSAP1	**BUB1B**	TOP2A
14	MELK	**BUB1B**	UBE2C	NUSAP1	ASPM	**BUB1B**	KIF23	NCAPH	**BUB1B**	ASPM	**BUB1B**
15	KIF20A	NCAPH	KIF20A	BIRC5	HJURP	ZWINT	BIRC5	ZWINT	NCAPH	UBE2C	BIRC5
16	AURKB	NCAPG	AURKB	KIF23	PRC1	PRC1	**BUB1B**	KIF23	KIF23	KIF23	CKAP5
17	**BUB1B**	KIF20A	KIF23	TTK	NCAPH	CCNB2	KIF20A	NCAPG	BIRC5	KIF20A	ESPL1
18	PRC1	CDK1	BIRC5	**BUB1B**	KIF20A	KIF23	ASPM	BIRC5	KIF20A	NCAPH	MCM3
19	NCAPG	BIRC5	CDC20	ZWINT	NCAPG	BIRC5	CDK1	CENPF	TTK	PRC1	CCNA2
20	BIRC5	CENPF	TTK	NCAPG	CCNB2	KIF20A	NCAPG	CDK1	PRC1	CDC20	CCNB2
21	CDKN3	CDCA3	NCAPG	AURKB	CCNB1	CDC20	AURKB	KIF20A	CDCA3	CDKN3	NDC80
22	PTTG1	CDC20	CENPF	PRC1	TTK	NCAPG	ECT2	TTK	CDK1	CDK1	XPO1
23	CDC20	AURKB	PRC1	KIF20A	AURKB	CDCA8	AURKA	PRC1	AURKB	PTTG1	CENPA
24	CCNA2	CDKN3	NDC80	CDK1	PTTG1	PTTG1	ZWINT	AURKB	CDC20	CCNB1	BRCA1
25	TOP2A	KIF23	CDCA3	CDC20	CDC20	AURKB	PRC1	CDC20	CEP55	TTK	CENPE

d1–d10: ten datasets sampled from the modeling datasets.

### GO and pathway enrichment analysis of blue and yellow module genes

To explore the biological functions of the blue and yellow modules, we performed Gene Ontology (GO) term enrichment analysis, as well as pathway ontology analyses from the KEGG, BIOCARTA, and REACTOME databases. All significant terms enriched in the above annotation systems are represented as a word cloud to facilitate comparison of the relative significance of enriched terms, where the grayscale and font size of each term are proportional to the adjusted *p value* derived from the enrichment analysis. For the blue module, the top enriched terms in GO and REACTOME pathway ontology were “extracellular matrix (ECM) organization” or “ECM structural constituent”. On KEGG pathway analysis, the top enriched terms were “focal adhesion” (adjP = 6.9e-11) and “ECM-receptor interaction” (adjP = 1.0e-10) (Figure 2B).

For yellow module genes, the top enriched terms in the GO and pathway databases were “mitotic cell cycle,” “DNA binding or replication” and “condensed chromosome kinetochore” (Figure 3B). Thus, the enriched terms in the annotation systems were mostly related to mitosis. These findings corroborate previous research implicating extensive cell proliferation and accelerated DNA replication as fundamental characteristics of tumor cells.

### Validation of the robust correlation between blue module eigengene expression and SOC stages

**Figure 4:**
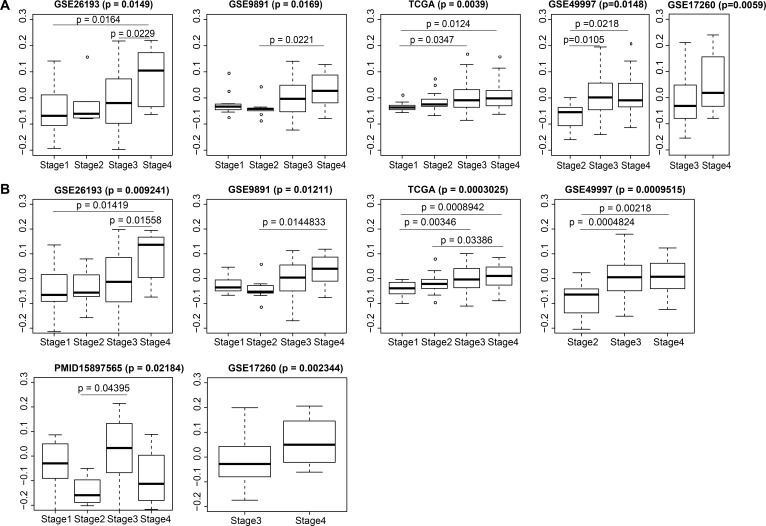
Distributions of blue module eigengene expression among traits in modeling and validation datasets Overall *p*-values and pairwise *p* values are shown. (**A**) 884 genes; (**B**) top 7 genes.

For a more intuitive depiction of the the expression distribution of module genes related to SOC stages, we calculated statistical significance via Kruskal-Wallis tests and plotted the module eigengene expression distribution for stages in each modeling dataset (i.e. GSE26193, GSE9891, and TCGA). For the above cases, eigengene expression showed significant differences between stages [Benjamini-Hochberg (BH) adjusted *p* < 0.05]. Meanwhile, positive correlations between eigengene expression and stages were universally demonstrated in all boxplots (Figure 4).

Since this co-expression network was identified in three public datasets and the correlation of its eigengene expression with stages in each dataset was validated, we determined if this correlation would be a universal rule across SOCs by perusing the other five independent SOC datasets from the curatedOvarianData package (GSE49997, GSE17260, TCGA.RNASeqV2, GSE20565, and PMID15897565). General information for the eight modeling or validation datasets examined is shown in Table 1. We calculated the eigengene expressions of module genes in these five validation datasets, and estimated the expression distribution among different stages using nonparametric tests. The distribution, mean value, and statistical results are shown in Figure 4A. From the boxplot, we found that the eigengene expression of the blue module genes showed a statistically significant distribution within stage III and stage IV patients in GSE17260 (*p* = 0.0059) and GSE49997 (*p* = 0.0148). There were significant differences between stage II and stage III (*p* = 0.0105), as well as between stage II and stage IV (*p* = 0.0218). In the other three datasets, the *p* values were greater than 0.05 (data not shown). As fewer numbers of module genes are likely needed for clinical transformation, we attempted to use the top seven hub genes to replace the 884 blue module genes. These hub genes included MMP2, COL3A1, COL1A2, FBN1, COL5A1, COL5A2, and AEBP1. There were significant differences (with lower *p* values) between eigengene expression of the top seven hub genes and tumor stages in the three modeling datasets (*p* = 0.0003025–0.01211) and in four validation datasets (*p* = 0.0009515–0.02184) (Figure 4B).

### Validation of the robust correlation between yellow module eigengene expression and SOC grades

**Figure 5:**
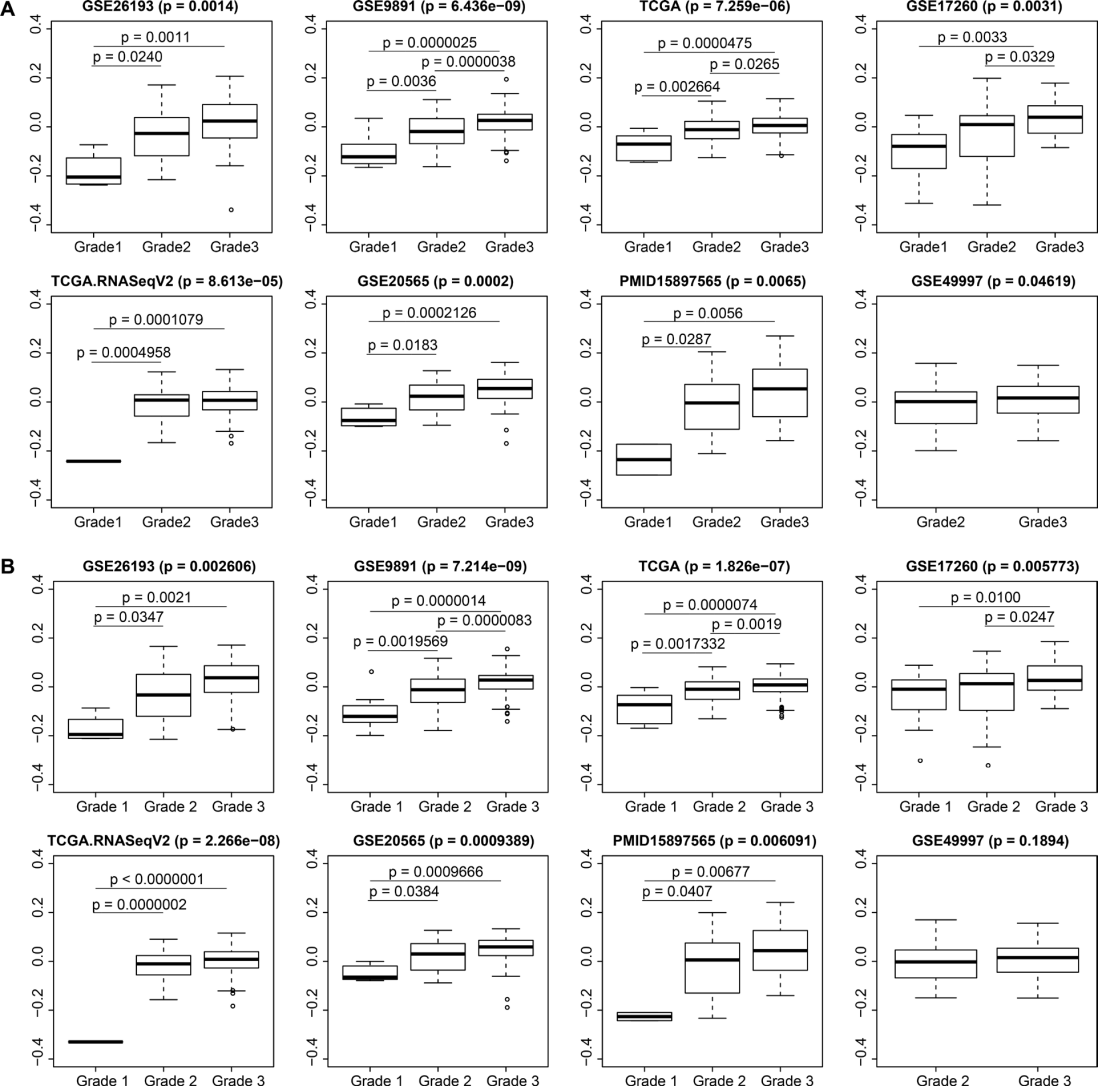
Distributions of yellow module eigengene expression among traits in modeling and validation datasets Overall *p*-values and pairwise *p* values are shown. (**A**) 561 genes; (**B**) top 7 genes.

Significant differences were found between the yellow module eigengene expression values and different tumor grades in all modeling and validation datasets. Similarly, positive correlations between eigengene expression and tumor grades were demonstrated in all boxplots. There were significant differences between any pair of grade 1, grade 2, and grade 3 SOC in GSE9891 (*p* = 6.43e-09) and TCGA (*p* = 7.259e-06). Meanwhile, in GSE26193 (*p* = 0.0014), GSE20565 (*p* = 0.0002), TCGA.RNASeqV2 (*p* = 8.613e-05), and PMID15897565 (*p* = 0.0065), differences appeared between grade 1 and grade 2, as well as between grade 1 and grade 3. Additionally, significant differences in eigengene expressions between grade 2 and grade 3 tumors were found in GSE17260 (*p* = 0.0329) and GSE49997 (*p* = 0.04619). We next used the top seven hub genes: CDK1, BUB1, BUB1B, BIRC5, AURKB, CENPA, and CDC20 to replace 561 yellow module genes. There were significant differences between the eigengene expression values of the top seven hub genes and tumor grades in the three modeling datasets and in four validation datasets (Figure 5B).

## DISCUSSION

In this study we integrated large-scale transcriptional profiling, incorporating three modeling datasets with 788 SOC samples, to identify robust co-expression modules associated with cancer characteristics. Our long-term goal was to provide insights into disease biology and diagnostic classification, which may cover the shortage of objectivity in postoperative pathological diagnosis and guide early-phase clinical therapeutic applications. We also determined that co-expression networks reflect causative relationships between gene-gene interactions. First, this study constructed two SOC-stage-specific (blue and ivory) and two grade-specific (yellow and white) modules based on ten random datasets sampled from 788 SOC samples. Second, we identified the shared hub genes in these ten datasets and found mutual subnetwork hub genes from the high-quality STRING protein interaction database for the blue and yellow modules. Third, we illustrated hub gene interactions and performed gene enrichment analysis on GO and pathway terms. Extracellular matrix organization genes were enriched for stage-related modules (blue), while cell cycle genes were enriched for grade-related modules (yellow). Then, we validated the correlations between module eigengene expression and tumor stages or grades in modeling datasets and other public validation datasets that were not used to build co-expression networks but showed ideal robustness.

The top hub genes within the blue module included FBN1, COL5A1, COL5A2, and AEBP1. A gene intersection set of the top 25 hub genes among the ten random sampling datasets and the high-quality STRING database contained MMP2, COL3A1, and COL1A2. The last two collagen (COL) proteins interacted with each other directly and with MMP2 through COL16A1. According to previous studies on ovarian cancer biomarkers, FBN1 and MMP2 were found to be metastasis-promoting markers that were stimulated or suppressed by Aurora-A or BRCA2. Clinically, high expression of FBN1 indicated poor disease-free survival [19] and overall survival [20]. COL5A1 and AEBP1 were also reported as metastatic signatures associated with poor overall survival in SOC [21]. The expression of COL3A1, COL5A2, and COL1A2 was also studied by immunocytochemistry and western blot analysis and found to be associated to drug-resistance in ovarian cancer [22]. ECM-receptor interaction was enriched based on the KEGG pathway in our study, while COL3A1, COL5A2, and COL2A1 were regarded as potential ECM components associated with cytostatic drug resistance in ovarian cancer cells [23]. Consequently, both top hub genes and intersection set genes all had close relationships with ovarian cancer, however, the exact roles of these hub genes in ovarian tumorigenesis, metastasis or drug resistance remain unknown. Our gene module co-expression network may provide clues to the complex regulatory networks between these various molecular components. Additionally, the blue module was related to different tumor stages and its eigengene expression values can be utilized as a more objective staging system to improve current clinical-pathological staging systems.

The gene intersection set of the yellow module hub genes and the high-quality STRING database contained CDK1, BUB1, BUB1B, BIRC5, AURKB, CENPA, and CDC20. The last six genes interacted with each other directly, while CDK1 interacted with CENPA through FOXM1. Cytoplasmic CDK1 overexpression was correlated with cancer growth and poor overall survival in 249 EOCs [24]. CDK1 was also found to be a potential target of transcription factors to regulate paclitaxel resistance in EOC patients [25]. While a few EOC studies implicated BUB1, BUB1B, BIRC5, AURKB, CENPA, and CDC20, multiple studies in contrast identified aberrantly increased expression of FOXM1 and its regulatory factors [26–29]. Overexpression of BUB1 was reported in non-small cell lung cancer [30] and breast cancer [31]. TPX2 (Targeting Protein for Xklp2), CENPA, and KIF4A were the top three hub genes in most of the ten sets. Although the function of TPX2 in EOC pathology is unknown, it was reported as a biomarker of poor survival in 143 EOC patients [32]. In cervical cancer, the expression of TPX2 was correlated with histological grading, FIGO staging, and lymph node metastasis [33]. Additionally, TPX2 was verified as a target gene of microRNA-491 in esophageal cancer and played a critical role in cancer cell invasion in both esophageal [34] and colon cancer [35]. Moreover, TPX2 was found to be a binding partner and activator of AURKA [36]. KIF4A, on the other hand, is critical for mitotic regulation, including chromosome condensation, spindle organization, and cytokinesis [37]. Abnormal expression of KIF4A induced apoptosis in breast cancer [38] and metastatic invasion in lung cancer [39]. In summary, compared with blue module hub genes, much less is known about the molecular actions of the yellow module hub genes. Research on these hub genes is imperative to fully uncover how alterations in cell differentiation relate to SOC.

Due to the high heterogeneity of gene expression profiles, it is more difficult to find shared co-expression networks across databases of ovarian cancers than it is for other types of tumors. Although some significant modules were detected among very few datasets, these modules may not provide accurate information on actual biological characteristics of tumors. In one network analysis of ovarian cancer, COL5A2, TPX2, and BIRIC5 were also identified as hub genes using the joint sparse regression model [40]. Another study built a Bayesian network model, which used only the TCGA dataset and 68 seed genes reported in the literature [41]. In another publication, WGCNA was performed based on TCGA RNA-sequencing data and only studied TP53 mutations [15]. To the best of our knowledge, our study is the first to construct a thorough and weighted co-expression network analysis of gene expression relationships with prognostic factors and outcome. Moreover, 788 SOC patients were used to build the WGCNA model and another 778 patients were used to validate it. Since the yellow module eigengene expression showed a negative correlation with cell differentiation and its distribution showed significant differences among distinct tumor grades, we conclude that there is some regulatory causality between hub genes expression profiles and tumor grades. This is relevant, inasmuch as high-grade SOCs entail significantly higher risk of death than low-grade ones, while the existing grading system does not reliably differentiate grade 2 from grade 1 or grade 3 patients. We speculated that hub gene expression could be used to represent a true continuum from grade 1 to 3 and reliably divide grade 2 SOCs into low-grade and high-grade groups. Our future work will further refine and validate the above classification method in multicenter prospective studies including SOC patients with various tumor grades.

## MATERIALS AND METHODS

### Datasets filtering

The ovarian cancer datasets were adopted from the curatedOvarianData Bioconductor package [16]. This package represents a manually curated data collection for gene expression meta-analysis of patients with ovarian cancer, and includes both uniformly prepared microarray data and curated and documented clinical metadata. According to our special rules, only datasets and samples that contain tumor stage and grade information, as well as survival data (recurrence status, vital status, recurrence time, and vital time) were reserved. Accordingly, GSE17260, TCGA.RNASeqV2, and GSE49997 datasets were excluded from the validation sets due to absence of stage I or stage II patient data. GSE20565 and PMID15897565 were added into the validation sets as they contained complete grade and stage information. All the datasets were originated from both microarrays platforms (hthgu133a, hgu133plus2, and hgug4112a) and RNA-seq technologies. We finally kept three modeling and five validation datasets (Table 3). To adapt to gene co-expression network analysis, only the common genes in all 3 modeling datasets were kept. Adjustment for batch effects was performed using the ComBat method [42], and 788 patients were randomly sampled to form ten datasets, extracting half of the total samples each time. These ten sets were used to construct a gene co-expression network.

### Gene co-expression network construction

Gene co-expression network analysis was performed using the R package WGCNA [9]. The process is summarized as follows. First, a matrix of pairwise correlations between all pairs of genes across all selected samples was constructed. Second, we chose 3 as the proper soft-thresholding power to which co-expression similarity is raised to achieve consistent scale-free topology in multiple datasets (Figure 1A). Third, with the chosen power value, we performed automatic network construction and module detection with the following major parameters: maxBlockSize of 20000, minModuleSize of 40, deepSplit of 4, and mergeCutHeight of 0.25. This procedure comprised calculation of network adjacencies and topological overlap dissimilarities, followed by scaling of topological overlap matrices and calculation of consensus topological overlap. Then, we built a hierarchical clustering dendrogram of gene expression data for each dataset, and performed adaptive branch cutting to identify shared functional modules. Some modules with similar expression profiles were merged, according to pre-defined parameters (Figure 1B) [43].

### Calculation of module-trait correlations and module preservation

Next, we determined correlations among gene expression modules and clinical traits for each of the above ten data sets. Tumor stage and grade, as well as survival data (recurrence status, vital status, recurrence time, and vital time) were chosen as clinical traits. Modules having significant relationships with one or more traits are shown in Supplementary Figure 1. After the above procedures, modules shared in ten or nine sets were detected (Figure 1C–1E). Modules were labeled using a conventional color scheme.

A WGCNA integrated function (modulePreservation) was used to calculate module preservation statistics and the Z summary score (Z score) was applied to evaluate whether a module was conserved or not [19].

### Calculation of eigengene expression

In the co-expression network, the first principal component (PC) of each module's gene expression matrix is referred to as the module eigengene (ME), a single value that represents the highest percent of variance for expression values for all module genes in a sample [44]. Thus, the expression profiles of module genes can be summarized as the expression profile of MEs. For convenience, ME expressions were used to discuss the correlation of gene expression modules with clinical traits. Moreover, module similarities can be measured with MEs, and some modules with high similarities can be merged according to a predefined threshold. Expression distribution differences in ME among multiple tumor grades or stages were calculated via nonparametric Kruskal-Wallis tests based on a null distribution inferred from permutation. If statistical significance existed among grades or stages, pairwise differences were estimated by Tukey's HSD tests, and pairwise *p* values adjusted by the BH method (Figures 4, 5).

### Hub gene identification

To evaluate the interactions of module genes and identify hub genes in each dataset, we calculated their in-module connectivity from the scale-free, weighted gene co-expression networks established in the ten sets as defined above. The connectivity of one node was defined as the sum of correlation coefficients with other nodes in a ‘signed’ topological overlap matrix (TOM) based on an adjacency matrix. For illustration purposes, we only extracted the top 200 strongest connections within the blue and yellow modules from each set to show the distribution of hub genes (Supplementary Figures 2 and 3).

Furthermore, we extracted a subnetwork with module genes from the high quality STRING protein interaction database (combined score ≥ 600) (Figures 2A and 3A) [45]. Since the STRING database weights and integrates information from numerous sources, including experimental repositories, computational prediction methods and public text collections, we only parsed the high-quality part of it, hoping to get a convincing interaction subnetwork of our module genes. The subnetwork was illustrated with Gephi [46].

### Enrichment analysis

Enrichment analysis was based on either Fisher's exact test or hypergeometric test using the R package dnet [47]. We employed the hypergeometric test to estimate enrichment significance in our modules, and the *p* values were batch-adjusted with the BH method. All the enriched terms are represented as word clouds, whit font grayscales and sizes proportional to their enrichment significance [48] (Figures 2B and 3B).

## SUPPLEMENTARY FIGURES AND TABLES






