# Supplementary material for: Gene co-expression network reveals shared modules predictive of stage and grade in serous ovarian cancers
Source: Oncotarget. 2017 May 11;8(26):42983–96. doi: 10.18632/oncotarget.17785 (PMC5522121; doi:10.18632/oncotarget.17785)
Supplement: Supplementary file 1 [file oncotarget-08-42983-s001.pdf]

Gene co-expression network reveals shared modules predictive of stage and grade in serous ovarian cancers

Supplementary Materials

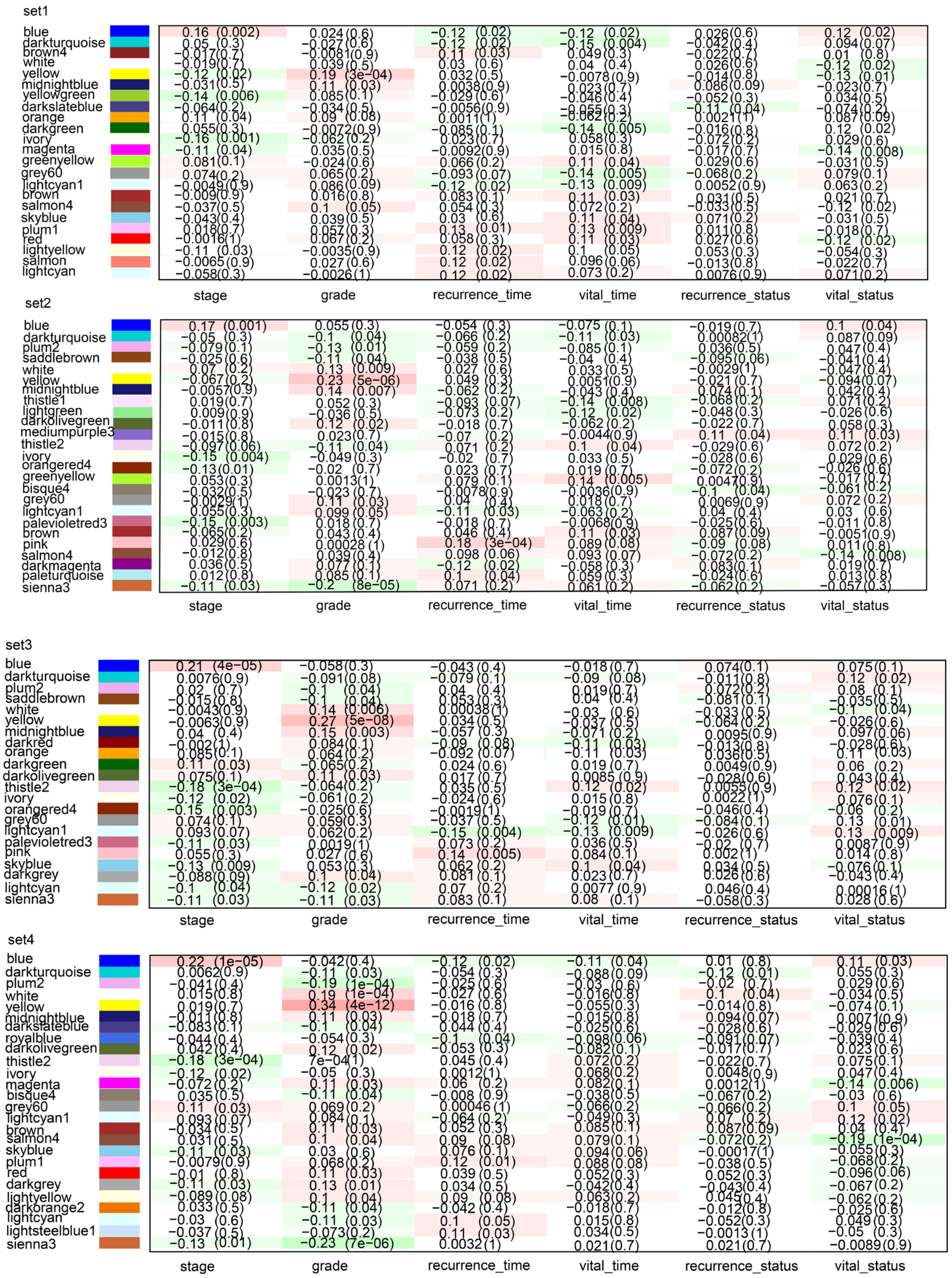

set5

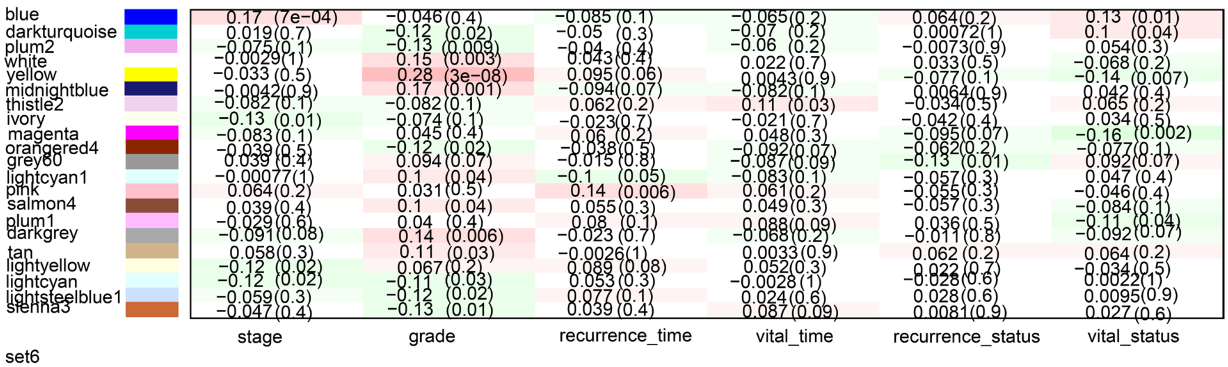

set6

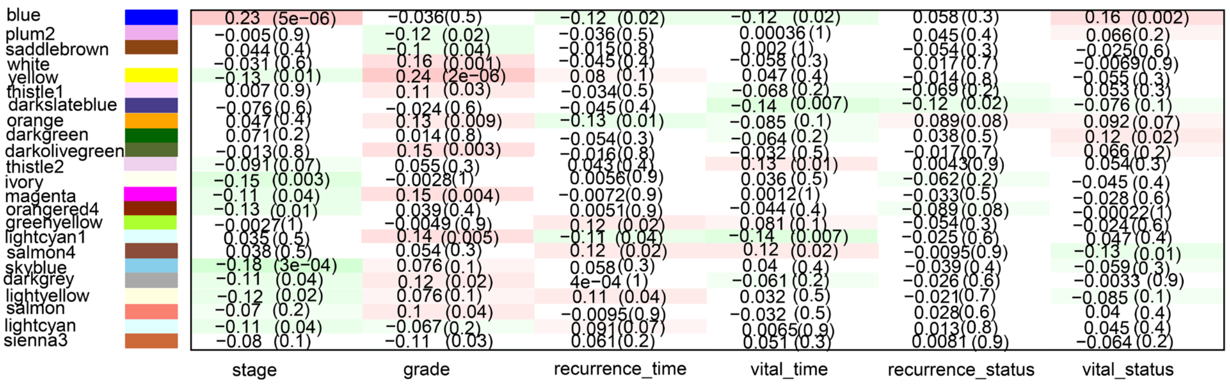

set7

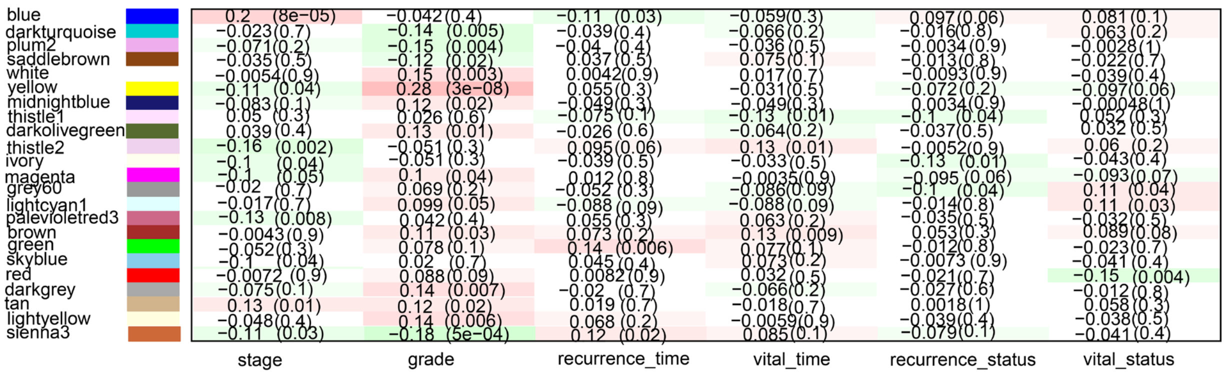

set8

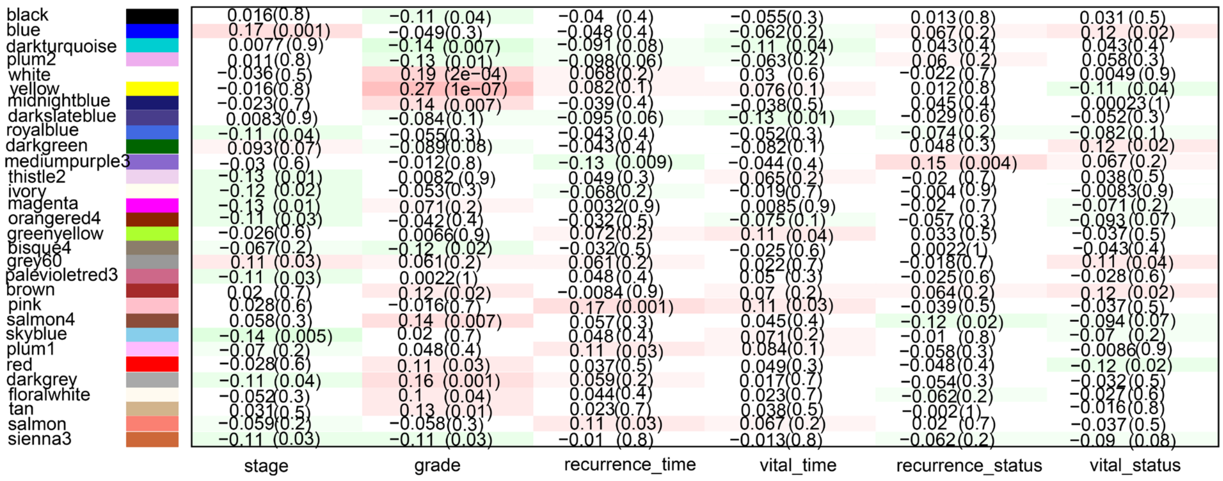

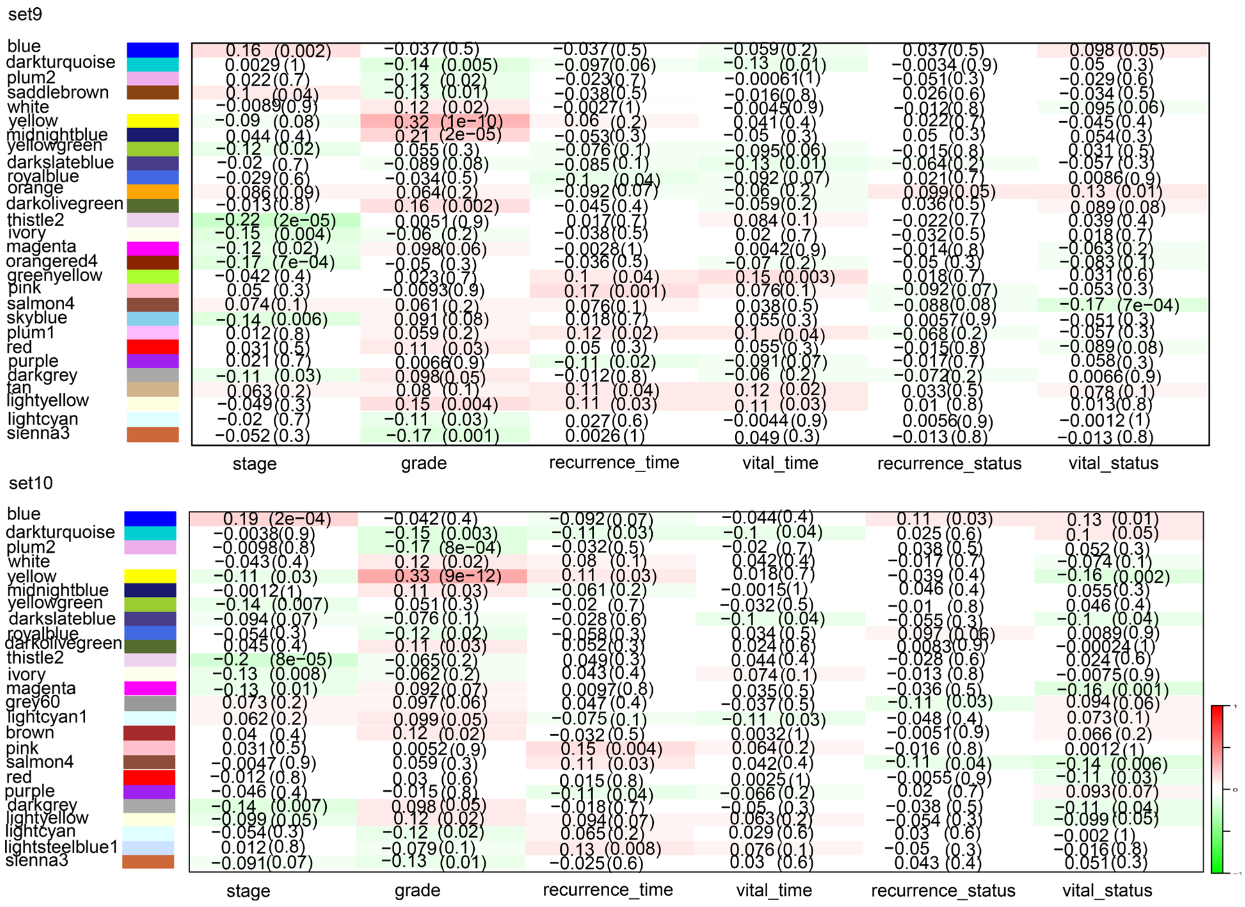

Supplementary Figure 1: Module-trait relationships across ten random sampling datasets. Correlation values and *p* values (in parenthesis) of module-trait relationships were demonstrated. Each row represented the module named after its color and each column represented the traits, containing tumor stage, grade, recurrence time, vital time, recurrence status and vital status. Any modules showed significant relationships with one or more traits were demonstrated (*p* value < 0.05).

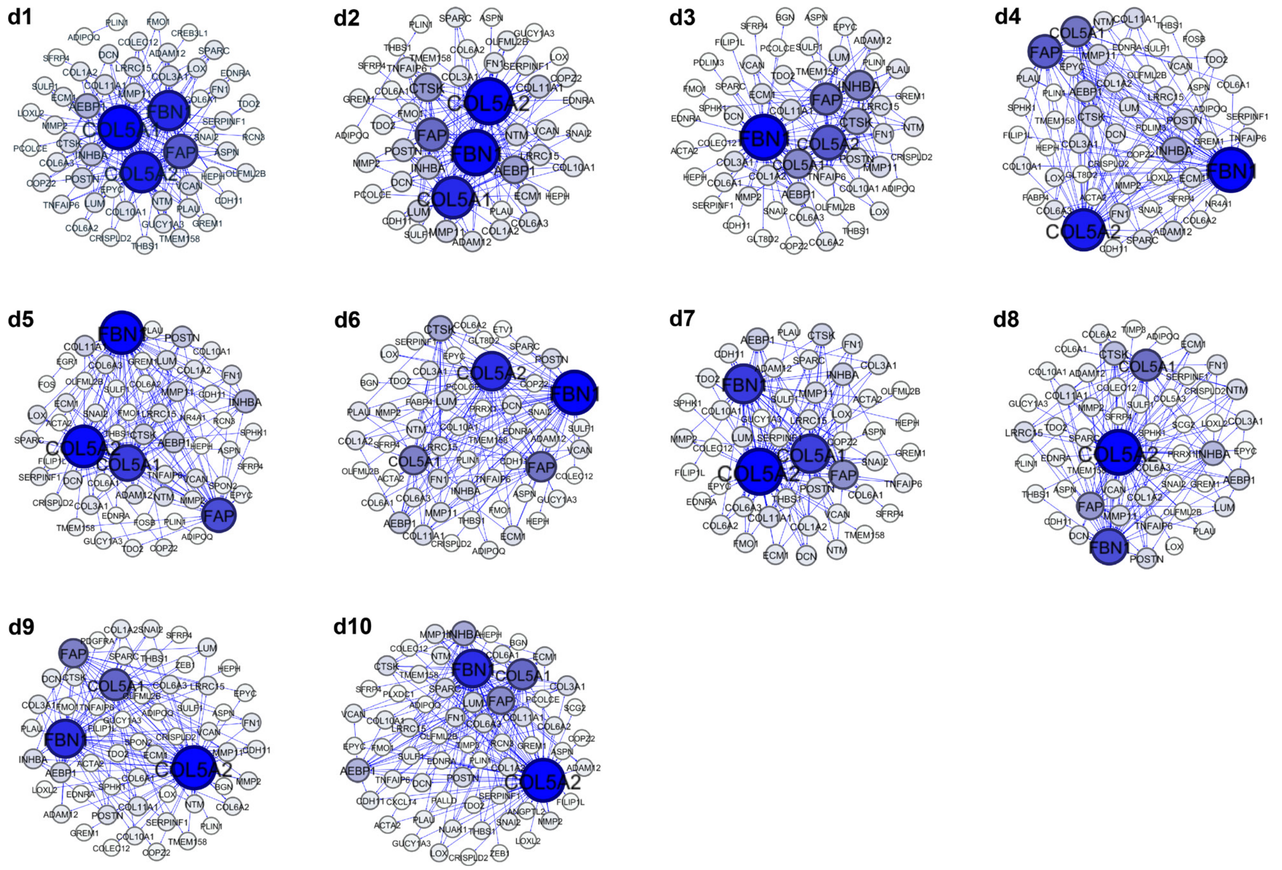

Supplementary Figure 2: The blue module gene network of top 200 hub genes in ten datasets. Hub genes of module were shown in blue, gene importance were according to the diameter of circle and color depth in descending order.

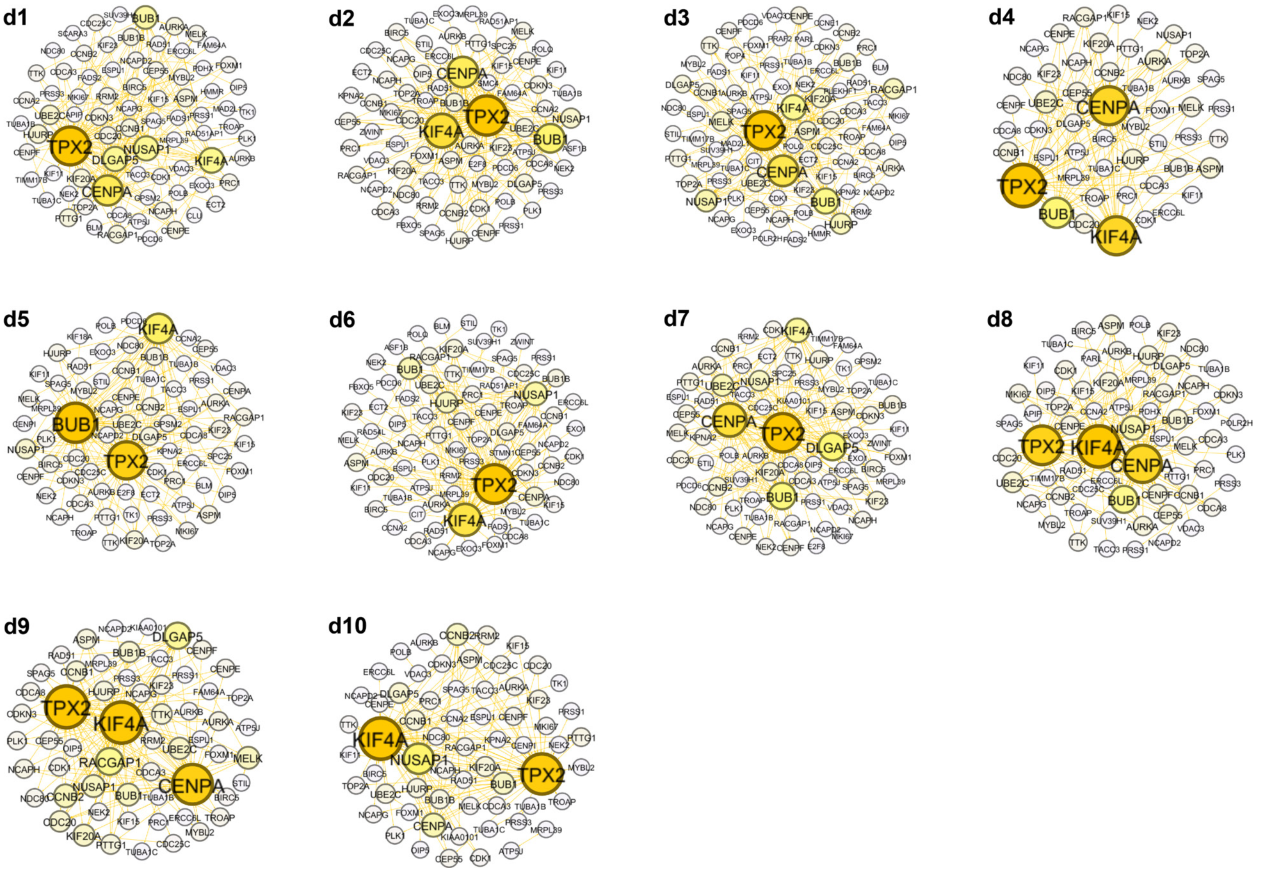

Supplementary Figure 3: The yellow module gene network of top 200 hub genes in ten datasets. Hub genes of module were shown in yellow, gene importance were according to the diameter of circle and color depth in descending order.

Supplementary Table 1: module preservations of all significant modules across ten datasets. See Supplementary_Table_1

Supplementary Table 2: Gene names of four meaningful modules. See Supplementary_Table_2
