# Supplementary material for: Gene co-expression network reveals shared modules predictive of stage and grade in serous ovarian cancers
Source: Oncotarget. 2017 May 11;8(26):42983–96. doi: 10.18632/oncotarget.17785 (PMC5522121; doi:10.18632/oncotarget.17785)
Supplement: Supplementary file 2 [file oncotarget-08-42983-s002.docx]

**Table S1 module preservations of all significant modules across ten datasets.**

| Number | Module Color | Gene Numbers | Z score |
| --- | --- | --- | --- |
| 1 | turquoise | 2565 | 84.13065 |
| 2 | blue | 884 | 78.82432 |
| 3 | yellow | 561 | 61.51173 |
| 4 | brown | 675 | 55.65162 |
| 5 | tan | 121 | 50.50167 |
| 6 | darkred | 89 | 44.42115 |
| 7 | magenta | 126 | 43.54587 |
| 8 | green | 484 | 43.33201 |
| 9 | red | 436 | 42.12788 |
| 10 | plum1 | 61 | 40.23722 |
| 11 | sienna3 | 64 | 40.10577 |
| 12 | black | 130 | 39.73008 |
| 13 | greenyellow | 122 | 37.72627 |
| 14 | lightcyan1 | 53 | 37.66333 |
| 15 | midnightblue | 105 | 36.6307 |
| 16 | lightgreen | 96 | 36.45626 |
| 17 | lightcyan | 97 | 36.31189 |
| 18 | pink | 130 | 35.89772 |
| 19 | yellowgreen | 62 | 35.30522 |
| 20 | plum2 | 45 | 35.03182 |
| 21 | skyblue | 75 | 34.04213 |
| 22 | darkgray | 82 | 33.66859 |
| 23 | darkturquoise | 83 | 33.57805 |
| 24 | purple | 124 | 33.1335 |
| 25 | lightsteelblue1 | 54 | 33.12725 |
| 26 | salmon | 118 | 32.89475 |
| 27 | gray60 | 97 | 32.5625 |
| 28 | paleturquoise | 73 | 32.55285 |
| 29 | royalblue | 91 | 32.27131 |
| 30 | darkmagenta | 66 | 32.19467 |
| 31 | orange | 82 | 31.86253 |
| 32 | brown4 | 48 | 31.77179 |
| 33 | darkorange | 81 | 31.32338 |
| 34 | cyan | 110 | 31.27757 |
| 35 | white | 78 | 30.30423 |
| 36 | orangered4 | 59 | 30.26807 |
| 37 | gray | 1169 | 30.01075 |
| 38 | saddlebrown | 75 | 29.34335 |
| 39 | floralwhite | 50 | 28.62533 |
| 40 | steelblue | 74 | 28.55528 |
| 41 | lightyellow | 94 | 27.8828 |
| 42 | salmon4 | 43 | 27.69909 |
| 43 | ivory | 52 | 27.40992 |
| 44 | darkslateblue | 46 | 27.36529 |
| 45 | darkorange2 | 50 | 26.75284 |
| 46 | thistle2 | 45 | 26.48972 |
| 47 | mediumpurple3 | 58 | 25.39137 |
| 48 | bisque4 | 47 | 25.13539 |
| 49 | palevioletred3 | 42 | 25.1011 |
| 50 | skyblue3 | 62 | 24.67649 |
| 51 | thistle1 | 44 | 23.14225 |
| 52 | violet | 70 | 22.96688 |
| 53 | darkgreen | 87 | 19.15775 |
| 54 | darkolivegreen | 67 | 17.02409 |
