# Supplementary material for: Gene co-expression network reveals shared modules predictive of stage and grade in serous ovarian cancers
Source: Oncotarget. 2017 May 11;8(26):42983–96. doi: 10.18632/oncotarget.17785 (PMC5522121; doi:10.18632/oncotarget.17785)
Supplement: Supplementary file 3 [file oncotarget-08-42983-s003.docx]

**Table S2 Gene names of four meaningful modules.**

| Module Color | Gene Name |
| --- | --- |
| Blue | ABCA1 / ABCA6 / ABCA8 / ABL2 / ABR / ACADM / ACAN / ACO1 / ACSBG2 / ACSM3 / ACTA2 / ACTG2 / ACTN1 / ACTR2 / ACVR1 / ACVR2A / ACYP2 / ADAM12 / ADAM19 / ADAM9 / ADAMTS12 / ADAMTS2 / ADAMTS5 / ADARB1 / ADH1B / ADH7 / ADIPOQ / ADRA2A / AEBP1 / AFAP1 / AFG3L2 / AFTPH / AGTR1 / AK5 / AKAP12 / AKAP13 / AKR1C4 / AKT3 / ALDH1A1 / ALDH1A3 / ALDH1B1 / ALDH1L1 / ALDH5A1 / ALLC / AMIGO2 / AMPH / ANGPT1 / ANGPTL2 / ANTXR1 / ANXA2 / ANXA5 / ANXA6 / AOC3 / AOX1 / APBB2 / APCS / APLNR / APOD / APOLD1 / AQP1 / ARFGAP3 / ARHGAP10 / ARHGAP24 / ARHGAP28 / ARHGAP6 / ARL15 / ARMC4 / ARMCX3 / ARSJ / ARTN / ASPH / ASPN / ATF3 / ATP10A / ATP10D / ATP12A / ATP2B2 / ATP5G3 / ATXN7L1 / AXL / AZI2 / BAG2 / BAG3 / BARX2 / BASP1 / BCHE / BCL11A / BCL2L14 / BDH1 / BDKRB1 / BDKRB2 / BGN / BHLHE40 / BICC1 / BMP1 / BMP2 / BMP4 / BMPR2 / BNC2 / BTG2 / BTG4 / C10orf95 / C11orf24 / C1orf115 / C1QTNF1 / C1QTNF3 / C5orf28 / C9orf3 / CA8 / CACNA1F / CALB2 / CALCRL / CALD1 / CALU / CAPN9 / CASK / CAV1 / CCDC102B / CCDC15 / CCDC88A / CCL21 / CD248 / CD34 / CD36 / CD93 / CD99 / CDC42EP3 / CDH11 / CDH12 / CDH5 / CDH6 / CDK14 / CDKL1 / CDKN1A / CDR2 / CFH / CH25H / CHN1 / CHPF / CHRD / CHRNA1 / CHST15 / CHSY1 / CIDEA / CILP / CKMT2 / CLCN2 / CLDN16 / CLDN5 / CLEC11A / CLIC3 / CLIC4 / CLIP2 / CLNS1A / CLSTN2 / CNGA1 / CNN1 / CNN3 / CNTN1 / COL10A1 / COL11A1 / COL15A1 / COL16A1 / COL1A2 / COL21A1 / COL3A1 / COL4A1 / COL4A2 / COL5A1 / COL5A2 / COL5A3 / COL6A1 / COL6A2 / COL6A3 / COL8A1 / COL8A2 / COLEC12 / COMP / COPZ2 / CORIN / COX5B / COX7A1 / CPA3 / CPE / CREB3L1 / CRISPLD2 / CRYGC / CSGALNACT2 / CST6 / CTGF / CTSK / CXCL12 / CXCL14 / CYB5R3 / CYP1A2 / CYP1B1 / CYP26A1 / CYP7B1 / CYR61 / CYTH3 / DAAM2 / DACT1 / DAPK1 / DCHS1 / DCHS2 / DCLK1 / DCN / DDR2 / DENND5A / DFNA5 / DIO2 / DIP2C / DKK1 / DKK2 / DLC1 / DLG3 / DNAJB4 / DNM1 / DPT / DPYSL2 / DPYSL3 / DSC3 / DST / DUSP1 / DUSP2 / DUSP3 / DUSP5 / DYNLT3 / DYRK3 / EBF2 / ECHDC3 / ECM1 / ECM2 / EDIL3 / EDNRA / EFEMP1 / EFEMP2 / EGFL6 / EGR1 / EGR3 / EHD2 / EHD3 / EIF1 / EIF5A2 / ELK3 / ELN / ELOVL5 / EMCN / EMILIN1 / EMP1 / ENC1 / ENG / ENOX1 / ENPEP / ENPP1 / ENTPD3 / EPHB2 / EPS8 / EPYC / ERG / ESR1 / ETS2 / ETV1 / EVC / EVI5 / EVL / F13A1 / F2R / F3 / FA2H / FABP4 / FAIM2 / FAM114A1 / FAM198B / FAM83E / FAP / FASTK / FAT1 / FAT4 / FBLN1 / FBLN5 / FBN1 / FBXL7 / FBXO34 / FER / FERMT2 / FEZ1 / FEZ2 / FGF1 / FGF18 / FGF7 / FGGY / FHIT / FHL3 / FHL5 / FHOD3 / FILIP1L / FKBP14 / FLNA / FLRT2 / FLT1 / FMO1 / FN1 / FNDC3B / FOLR1 / FOS / FOSB / FOSL2 / FOXF1 / FOXF2 / FOXH1 / FOXN3 / FOXO1 / FRMPD1 / FSTL1 / FSTL3 / FZD1 / FZD7 / GABRA3 / GADD45B / GADD45G / GALNT1 / GALNT10 / GALNT3 / GAS1 / GAS7 / GBAS / GDF9 / GEM / GFPT2 / GFRA1 / GGT5 / GHR / GJA1 / GLI3 / GLS / GLT8D2 / GMPR / GNAL / GNG11 / GNMT / GOLM1 / GPC5 / GPR1 / GPR116 / GPR124 / GPR176 / GPR4 / GPR75 / GPR87 / GREM1 / GREM2 / GRP / GSTM4 / GTPBP10 / GUCY1A2 / GUCY1A3 / GULP1 / HABP4 / HAPLN1 / HAS1 / HAS2 / HBB / HBEGF / HEPH / HEY1 / HHLA3 / HIC1 / HIF1A / HIGD1B / HLX / HMGCS2 / HOMER2 / HOPX / HOXA1 / HOXA5 / HOXC6 / HSD17B3 / HSD17B6 / HSPA13 / HSPA2 / HSPB7 / HSPB8 / HSPG2 / HTR2A / HTRA1 / ID1 / ID2 / ID3 / IER2 / IGF1 / IGFBP4 / IGFBP6 / IGFBP7 / IL18R1 / IL1RAP / IL1RL2 / IL6 / IMPG2 / INHBA / INO80B / INPP5F / INPP5J / IRF6 / ISL1 / ISLR / ITGA5 / ITGB1 / ITGB5 / ITGBL1 / ITIH5 / JAM2 / JAM3 / JUN / JUNB / KAL1 / KANK2 / KCNC4 / KCND2 / KCNE4 / KCNJ8 / KCNK5 / KCNQ1 / KCTD14 / KDELC1 / KDELR3 / KDR / KERA / KHDRBS3 / KIAA1199 / KIAA1324 / KIFC3 / KLF2 / KLF4 / KLF6 / KLF9 / KLHL4 / KLRF1 / LAMA4 / LAMB1 / LBH / LEP / LEPRE1 / LEPREL2 / LGALS1 / LHFP / LIFR / LIMA1 / LIN7A / LIPT1 / LMCD1 / LMOD1 / LOX / LOXL1 / LOXL2 / LPAR1 / LPAR3 / LPAR4 / LPL / LPP / LRCH1 / LRP1 / LRP12 / LRRC15 / LRRC17 / LRRC32 / LRRN3 / LSAMP / LTBP1 / LTBP2 / LUM / LUZP1 / LXN / LYVE1 / MAB21L2 / MAN1C1 / MAP1A / MAPRE2 / MARCKS / MATN3 / MCAM / MCC / MDH2 / MED9 / MFAP4 / MFAP5 / MFGE8 / MGAT5 / MGP / MME / MMP1 / MMP11 / MMP13 / MMP14 / MMP19 / MMP2 / MMP3 / MN1 / MORC4 / MOXD1 / MRC2 / MRPL48 / MRPS33 / MSC / MTL5 / MTSS1 / MXRA7 / MYB / MYCT1 / MYH11 / MYH9 / MYLIP / MYLK / MYO15A / MYO1D / MYO1E / NACC2 / NAV3 / NCOR2 / NDEL1 / NDN / NDUFB2 / NEDD4 / NET1 / NFIL3 / NGFR / NID1 / NID2 / NKX3-2 / NOTCH1 / NOTCH4 / NOX4 / NPTX1 / NR4A1 / NR4A2 / NR4A3 / NRBF2 / NRBP1 / NRP1 / NRP2 / NT5E / NT5M / NTM / NUAK1 / NXF3 / OGN / OLFML1 / OLFML2A / OLFML2B / OMD / OR7A17 / OSBPL10 / OSBPL8 / OSR2 / OVGP1 / OVOL2 / P4HA1 / P4HA2 / PAAF1 / PAK1 / PAK6 / PAK7 / PALLD / PAMR1 / PANX1 / PAPSS2 / PARVA / PBX3 / PCDH12 / PCDH17 / PCOLCE / PCSK5 / PDE10A / PDE2A / PDE4D / PDE7B / PDGFC / PDGFD / PDGFRA / PDGFRB / PDGFRL / PDLIM2 / PDLIM3 / PDLIM5 / PDLIM7 / PDPN / PDZRN3 / PDZRN4 / PELI1 / PHACTR2 / PHLDA1 / PICALM / PIM1 / PITX2 / PKD2 / PLA2G2A / PLA2G3 / PLA2G5 / PLAGL1 / PLAT / PLAU / PLIN1 / PLK2 / PLN / PLOD1 / PLOD2 / PLS3 / PLSCR2 / PLVAP / PLXDC1 / PLXDC2 / PLXNA1 / PLXND1 / PMEPA1 / PMP22 / POLD2 / POM121 / POSTN / POT1 / PPAP2A / PPARGC1B / PPBP / PPEF1 / PPFIBP1 / PPIC / PPM1F / PPP1R12B / PPP1R13L / PPP1R14D / PPP1R15A / PPP1R3C / PPP3CA / PPP3CB / PRG4 / PRKACB / PRKCDBP / PRKCQ / PRKD1 / PRKG1 / PROCR / PROS1 / PRPH2 / PRRG1 / PRRX1 / PRSS21 / PRSS23 / PSD4 / PTGER3 / PTGFR / PTGIS / PTGS2 / PTHLH / PTPLA / PTPN13 / PTPN2 / PTPRB / PTPRD / PTPRG / PTPRM / PTRF / PVALB / QSER1 / RAB11FIP5 / RAB15 / RAB1A / RAB23 / RAB31 / RAB6B / RAI14 / RAMP3 / RAPGEF4 / RARA / RARRES2 / RBBP8 / RBMS3 / RBPMS / RCAN1 / RCN3 / RECK / RGS16 / RGS2 / RGS3 / RGS4 / RHOB / RHOBTB3 / RIN2 / RND3 / RNF128 / ROBO1 / ROBO4 / ROR2 / RORA / RORB / RORC / RPP38 / RTN4 / RUNX1 / RUNX1T1 / RUNX2 / RUSC2 / RYR2 / S100A10 / S1PR1 / SAMD4A / SAR1A / SATB1 / SATB2 / SCD / SCG2 / SCGB1D1 / SCGB1D2 / SCN3A / SCN7A / SCRG1 / SCUBE2 / SDC1 / SDC2 / SEC23A / SEC24D / SEC31A / SELENBP1 / SEMA3C / SEMA5A / 11-Sep / 4-Sep / 6-Sep / 7-Sep / SERPINB2 / SERPINE1 / SERPINF1 / SFRP1 / SFRP4 / SFXN3 / SH3BGRL / SH3PXD2A / SHC1 / SIK1 / SKAP1 / SKI / SKIL / SLC12A8 / SLC15A2 / SLC16A2 / SLC16A7 / SLC18A1 / SLC20A1 / SLC25A12 / SLC2A3 / SLC36A1 / SLC43A1 / SLC47A1 / SLC6A1 / SLC6A14 / SLIT2 / SLIT3 / SMAD6 / SMAD7 / SMPD1 / SMTN / SMYD3 / SNAI1 / SNAI2 / SNCAIP / SNTB2 / SOCS2 / SOCS3 / SORBS1 / SPAG9 / SPARC / SPARCL1 / SPDEF / SPHK1 / SPOCK1 / SPON2 / SPRY4 / SPSB1 / SRPK3 / SRPX / SRPX2 / ST7 / ST7L / ST8SIA1 / STARD13 / STARD7 / STK39 / STX2 / STYXL1 / SULF1 / SYDE1 / SYNC / SYNE1 / SYNJ2 / SYNM / SYTL2 / TAGLN / TBC1D8B / TBCEL / TBRG4 / TBX3 / TCF4 / TDO2 / TFF2 / TFPI / TGFB1I1 / TGFB3 / TGFBR2 / TGM2 / THBD / THBS1 / THRB / TIAM1 / TIE1 / TIMP1 / TIMP2 / TIMP3 / TLE4 / TMEM158 / TMEM2 / TMEM204 / TMEM45A / TMEM47 / TMPRSS3 / TNC / TNFAIP6 / TNFRSF12A / TNFSF4 / TPBG / TPM1 / TPM2 / TPM4 / TRAM2 / TRIB2 / TRIM10 / TRIM9 / TRPC1 / TRPC6 / TSC22D3 / TSPAN4 / TSPAN5 / TUBB2A / TUBB6 / TWIST1 / UAP1L1 / UBE2D1 / UGDH / UGT8 / UNC45A / UPK1B / VASH1 / VAT1 / VCAN / VCL / VEGFC / VGLL3 / VIM / VIP / VIPR1 / VKORC1 / VSNL1 / VWF / WBP5 / WDR3 / WDR41 / WDR70 / WDR77 / WIPI1 / WISP2 / WISP3 / WNT2 / WNT4 / WNT7A / WWC2 / WWC3 / XK / XYLB / XYLT1 / ZBTB3 / ZCCHC24 / ZEB1 / ZFHX4 / ZFP36 / ZFP36L1 / ZFPM2 / ZMAT3 |
| White | AKIRIN1 / ALG6 / ATP6V0B / BSDC1 / C1orf109 / C1orf123 / C1orf216 / C1orf50 / CPT2 / DPH2 / EBNA1BP2 / EIF2B3 / EIF3I / ELOVL1 / EPS15 / ERMAP / FAF1 / FOXJ3 / GNL2 / GPBP1L1 / HDAC1 / HECTD3 / HSPB11 / HYI / INPP5B / IPO13 / IPP / KDM4A / KIAA0319L / KPNA6 / LRRC41 / LRRC42 / MACF1 / MAST2 / MED8 / MRPS15 / MTF1 / MUTYH / NCDN / NDUFS5 / NFYC / NRD1 / OSBPL9 / PABPC4 / PHC2 / POMGNT1 / PPCS / PPIE / PPT1 / PRDX1 / PSMB2 / PTP4A2 / PTPRF / RLF / RNF11 / RNF220 / RRAGC / S100PBP / SCMH1 / SCP2 / SNIP1 / TESK2 / TMEM39B / TMEM53 / TMEM59 / TOE1 / TRAPPC3 / TRIM62 / TRIT1 / TXLNA / UROD / UTP11L / YARS / YIPF1 / YRDC / ZMPSTE24 / ZMYM1 / ZMYM4 |
| Yellow | ACAT2 / ACBD4 / ACOT7 / ACOX2 / ACSL3 / ACTL6A / AFF1 / AHNAK / ALDH3B1 / ALS2CL / AMMECR1 / ANP32E / ANXA9 / APEX2 / APH1B / APIP / APITD1 / APOOL / ARHGAP11A / ARHGAP33 / ARL6IP1 / ARNTL2 / ASF1B / ASPM / ATAD2 / ATAD5 / ATP5J / ATP6V1B1 / AURKA / AURKB / B3GALNT1 / BARD1 / BCAS2 / BCL2L1 / BCL2L11 / BCL6 / BIRC5 / BLM / BRCA1 / BRCA2 / BRD9 / BRIP1 / BRIX1 / BUB1 / BUB1B / BUB3 / C16orf59 / C17orf75 / C19orf40 / C1orf112 / C1orf116 / C5orf22 / C9orf40 / CABYR / CALCOCO1 / CAPN2 / CASC5 / CCNA2 / CCNB1 / CCNB2 / CCND1 / CCNE1 / CCNE2 / CCNF / CCT5 / CCT6A / CDC123 / CDC20 / CDC25A / CDC25B / CDC25C / CDC45 / CDC6 / CDC7 / CDCA3 / CDCA4 / CDCA8 / CDK1 / CDK2 / CDKN2A / CDKN3 / CDT1 / CENPA / CENPE / CENPF / CENPI / CENPJ / CENPM / CENPN / CENPO / CENPQ / CEP152 / CEP192 / CEP55 / CEP72 / CEP76 / CFL1 / CGGBP1 / CHAF1A / CHAF1B / CHD9 / CHEK1 / CHN2 / CHRNA5 / CIAO1 / CIRBP / CIT / CKAP2 / CKAP5 / CKS2 / CLIC5 / CLIP4 / CLN5 / CLU / CNTLN / CPD / CPSF6 / CREB3L2 / CRIM1 / CROT / CRTAP / CRY2 / CSTF2 / CYP27A1 / CYP3A5 / DAP / DARS2 / DBF4 / DCLRE1B / DCUN1D1 / DHFR / DHX29 / DIAPH3 / DLGAP5 / DMC1 / DMRT1 / DNAJB5 / DNAJC3 / DNAJC9 / DNMT1 / DNMT3B / DOCK9 / DONSON / DSN1 / DTL / DTYMK / E2F1 / E2F2 / E2F8 / EBP / ECT2 / ELAVL1 / ELF1 / ELOVL6 / EPB41L1 / ERAL1 / ERCC6L / ESPL1 / EWSR1 / EXO1 / EXOC3 / EXOG / EZH2 / FADS1 / FADS2 / FAM107A / FAM111A / FAM131A / FAM136A / FAM13A / FAM63A / FAM64A / FANCA / FANCC / FANCI / FANCL / FAR2 / FASTKD3 / FBXO5 / FEN1 / FLII / FMOD / FOXM1 / FOXRED2 / FRY / FXN / GAS6 / GDAP2 / GGCX / GGH / GINS2 / GINS3 / GINS4 / GIPC2 / GLMN / GMNN / GPR19 / GPSM2 / GTF3C5 / H1FX / H2AFV / H2AFX / H2AFZ / HELLS / HIST1H2AG / HIST1H2BH / HIST1H2BJ / HJURP / HMGB2 / HMGCS1 / HMMR / HN1 / HNRNPA2B1 / HNRNPM / HNRNPR / HS1BP3 / HSD11B2 / HSD17B10 / HSPA14 / IGBP1 / IKZF2 / IL20RA / IL6ST / IMPA2 / INCENP / INTS7 / IQSEC1 / ITCH / ITGA2 / ITGB3BP / ITGB4 / KCMF1 / KCND3 / KCNH2 / KCNMA1 / KCTD12 / KIAA0040 / KIAA0101 / KIF11 / KIF15 / KIF18A / KIF20A / KIF20B / KIF22 / KIF23 / KIF2A / KIF4A / KIN / KNTC1 / KPNA2 / KRT7 / LAMA3 / LAMB3 / LAMC2 / LBR / LHX2 / LMBRD1 / LMNB1 / LPCAT1 / LRP2 / LRRC20 / LRRC40 / LRRC8D / LY6G6C / MAD2L1 / MAGEC2 / MAGOH / 42435 / MCM10 / MCM2 / MCM3 / MCM4 / MCM5 / MCM6 / MCOLN3 / MCTP2 / MEF2D / MELK / MET / MFN1 / MGAM / MKI67 / MKKS / MLPH / MPPED2 / MRPL19 / MRPL39 / MSH2 / MSH6 / MSLN / MTF2 / MTHFD2 / MXD3 / MYBL2 / MYO19 / MYO3A / NAA38 / NASP / NCAPD2 / NCAPG / NCAPG2 / NCAPH / NDC80 / NDE1 / NDUFS6 / NEIL1 / NEIL3 / NEK2 / NEK7 / NF1 / NFAT5 / NMU / NNT / NPDC1 / NR1D1 / NR2C2 / NRAS / NRIP3 / NSMCE4A / NTS / NUDT1 / NUP155 / NUP160 / NUP205 / NUP210 / NUSAP1 / NXT2 / OIP5 / PAK3 / PAPD7 / PARL / PAX6 / PBK / PBXIP1 / PCNA / PDCD6 / PDE4A / PDHX / PDK1 / PDK3 / PDSS1 / PER2 / PERP / PGPEP1 / PGRMC1 / PHGDH / PHTF1 / PHTF2 / PIAS2 / PIN4 / PKMYT1 / PLA2G1B / PLA2R1 / PLCXD1 / PLEKHF1 / PLEKHG3 / PLK1 / PLK4 / PLLP / PLP2 / PLSCR4 / PMCH / PNP / PNRC1 / POLA1 / POLA2 / POLB / POLD1 / POLD3 / POLE / POLE2 / POLQ / POLR2D / POLR2H / PON2 / POP4 / PPL / PPP2R2B / PPP2R3B / PRAF2 / PRC1 / PRELP / PRIM1 / PRIM2 / PRNP / PRPS1 / PRSS1 / PRSS3 / PSMC3IP / PSMD1 / PSMD10 / PSMD11 / PSMD14 / PSME4 / PSMG2 / PSRC1 / PTBP2 / PTGER2 / PTPRN2 / PTTG1 / PURA / R3HDM1 / RAB17 / RACGAP1 / RAD1 / RAD51 / RAD51AP1 / RAD54B / RAD54L / RANGAP1 / RASAL1 / RASSF9 / RBBP7 / RBKS / RBL1 / RBL2 / RBM12 / RBM14 / RBM15 / RBM28 / REEP4 / RFC2 / RFC3 / RGL1 / RHOF / RHOT1 / RIF1 / RMI1 / RNASET2 / RPL15 / RPL39L / RPS6KA2 / RRAD / RRM1 / RRM2 / RSRC1 / SAC3D1 / SACS / SAP130 / SCARA3 / SERBP1 / SERINC3 / SFMBT1 / SFPQ / SH3YL1 / SHCBP1 / SIDT2 / SKA1 / SKP2 / SLC12A7 / SLC16A1 / SLC1A4 / SLC23A2 / SLC25A40 / SLC26A2 / SLC27A3 / SLC30A1 / SLC4A2 / SMC1A / SMC2 / SMC4 / SNRNP40 / SNRPA / SNRPB / SNRPD1 / SORBS2 / SOX9 / SPAG5 / SPATA20 / SPC25 / SPOCK2 / SRD5A1 / SSBP1 / SSRP1 / SSX2IP / ST5 / ST6GALNAC2 / STAT5B / STIL / STMN1 / SUB1 / SUV39H1 / SUV39H2 / SUZ12 / TACC3 / TADA2A / TAF5 / TANK / TARS / TFRC / TGM1 / TGOLN2 / TIMELESS / TIMM17B / TIPIN / TK1 / TK2 / TMEM106C / TMEM159 / TMEM183A / TMEM19 / TMEM194A / TMEM38B / TMEM97 / TMPO / TNFRSF11B / TOB2 / TOMM7 / TOP2A / TOPBP1 / TOR1AIP1 / TOR3A / TPM3 / TPX2 / TRA2B / TRAIP / TRIM29 / TRIO / TRIP12 / TRIP13 / TRMU / TROAP / TSHZ2 / TSPAN31 / TTF2 / TTK / TTLL7 / TUBA1B / TUBA1C / TUBA3C / TXNIP / UBE2C / UBL3 / UCHL1 / UCK2 / UNC5B / UPF3B / USP1 / UTP6 / VAMP4 / VANGL1 / VDAC3 / VRK1 / VRK2 / WDHD1 / WDR76 / XPO1 / XRCC3 / YBX2 / YPEL5 / ZBED2 / ZBTB20 / ZHX3 / ZIC1 / ZNF185 / ZNF200 / ZNF207 / ZNF35 / ZNHIT3 / ZWILCH / ZWINT |
| Ivory | ACBD3 / ADSS / AHCTF1 / AIDA / ANGEL2 / ARF1 / ARID4B / C1orf27 / C1orf35 / CDC42BPA / CDC73 / CNIH4 / COG2 / EGLN1 / EPRS / FBXO28 / FH / GGPS1 / GNPAT / GPATCH2 / IARS2 / JMJD4 / KCTD3 / KIAA0907 / MTR / NSL1 / NUP133 / NVL / PCNXL2 / PTPN14 / RAB3GAP2 / RAB4A / RCOR3 / RPS6KC1 / RRP15 / SCCPDH / SMYD2 / TAF1A / TAF5L / TARBP1 / TBCE / TFB2M / TIMM17A / TOMM20 / TRAF5 / TSNAX / TTC13 / UCHL5 / URB2 / WDR26 / ZNF672 / ZNF692 |
